# Consumer Acceptance of *Royal Gala* Apple Snacks Produced by Sun, Oven and Commercial Drying Methods: A Physicochemical and Sensory Perspective

**DOI:** 10.3390/foods15040762

**Published:** 2026-02-19

**Authors:** Lisete Fernandes, Pedro B. Tavares, José R. Fernandes, Alice Vilela, Fernando M. Nunes, Carla Gonçalves

**Affiliations:** 1 CQ-VR Centre of Chemistry-Vila Real, UME/CIDE Electron Microscopy Unit-Innovation and Development Centre, University of Trás-os-Montes and Alto Douro, 5000-801 Vila Real, Portugal; 2 CQ-VR Centre of Chemistry-Vila Real, UME/CIDE Electron Microscopy Unit-Innovation and Development Centre, Department of Chemistry, Universidade de Trás-os-Montes e Alto Douro, 5000-801 Vila Real, Portugal; 3 CQ-VR Centre of Chemistry-Vila Real, Department of Physics, University of Trás-os-Montes and Alto Douro, 5000-801 Vila Real, Portugal; jraf@utad.pt; 4 CQ-VR Centre of Chemistry-Vila Real, Department of Agronomy, University of Trás-os-Montes and Alto Douro, 5000-801 Vila Real, Portugal; avimoura@utad.pt; 5 CQ-VR Centre of Chemistry-Vila Real, Department of Chemistry, Food and Wine Chemistry Lab., University of Trás-os-Montes and Alto Douro, 5000-801 Vila Real, Portugal; fnunes@utad.pt; 6 CITAB—Centre for the Research and Technology of Agro-Environmental and Biological Sciences, University of Trás-os-Montes e Alto Douro, 5000-801 Vila Real, Portugal; carlagoncalves@utad.pt; 7 Epidemiology Research Unit, Laboratory for Integrative and Translational Research in Population Health, Institute of Public Health, University of Porto, 4050-600 Porto, Portugal

**Keywords:** *Royal Gala* dried apples, physicochemical properties, sensory attributes, CATA test

## Abstract

Drying conditions can markedly reshape the sensory and functional quality of fruit snacks and, ultimately, consumer acceptance. This study compares *Royal Gala* dried apple snacks produced by indirect sun drying (SDA), oven drying (ODA) and two commercial drying methods (CCA and CFA) using an integrated approach combining instrumental colour and texture analysis, sugar profiling, and the measurement of total phenolics and antioxidant activity along with the recording of consumer hedonic and descriptive responses. Consumers (n = 100) evaluated appearance, aroma, sweetness, texture, overall liking and consumption intention on a 9-point hedonic scale, which was complemented by attribute-selection frequencies. The drying method strongly affected colour development: the SDA samples exhibited the lowest browning index (96.78 ± 2.3) and the lightest colour (L* = 84.53), whereas the ODA, CCA and CFA samples showed progressively higher levels of browning (161.83 ± 3.5 to 194.10 ± 3.7). Total sugars ranged from 25.0 to 33.8 mg/100 g extract, with fructose predominating (≈52–69% of total sugars). Phenolic-related markers also differed significantly: the ODA sample presented with the highest total phenolic content (112.5 ± 2.6 mg GAE/100 g extract) and the SDA with the lowest (78.6 ± 1.9 mg GAE/100 g extract). DPPH inhibition was 75.7%, 71.7%, 68.4% and 63.9% for the SDA, ODA, CCA and CFA samples, respectively. ABTS results were consistent with this pattern, with the SDA sample also exhibiting high antioxidant activity (39.0 ± 2.1 μmol Trolox/g extract). Importantly, the SDA and ODA samples achieved the strongest consumer acceptance, with most participants assigning an overall liking score of 8/9, consistent with higher frequencies of favourable flavour and texture. Overall, the combined physicochemical–sensory evidence indicates that drying approach strongly impacts browning, sugar perception and bioactive-related functionality, with the SDA samples yielding the most preferred product profile among the tested dried apple snacks, outperforming industrial methods in terms of consumer acceptance.

## 1. Introduction

Apples (*Malus domestica* Borkh.) rank among the most consumed fruits worldwide and constitute a relevant model matrix for studying quality changes induced by processing [1]. In addition to broad availability and year-round supply, apples combine a sensory profile that consumers readily recognize (sweetness, mild acidity and characteristic aroma) with a nutritional composition that supports their positioning as a snack option [2,3,4]. Apples provide dietary fibre and a diverse set of bioactive compounds, notably phenolics, which contribute to their antioxidant capacity and have been associated with potential health-related effects in the context of regular fruit consumption [5,6,7]. From a product development perspective, apples are particularly suitable for dehydration because their structure yields a wide range of textures depending on the drying conditions and final moisture level, while intrinsic sugars and aroma-active compounds may be preserved or altered by heat and oxygen exposure [8,9,10]. The *Royal Gala* cultivar is widely commercialised and represents an especially relevant case due to its appealing sweetness and flavour balance and high consumer familiarity, which facilitates the detection of quality deviations and supports robust acceptance testing in snack formats [11,12,13]. Moreover, this cultivar is frequently used in commercial dried apple snacks, enabling direct comparison with market products that explicitly declare that they are derived from this cultivar.

Demand for convenient foods aligned with health-oriented choices has increased the visibility of fruit snacks, including dried fruit products, as alternatives to confectionery and highly processed snacks [14,15]. Within this sector, dried apple snacks hold a prominent position because they deliver sweetness and flavour without necessarily requiring added sugars, while offering portability and extended shelf-life [16,17]. Nevertheless, acceptance depends on more than the “health concept”, since consumers evaluate appearance, aroma, flavour authenticity, sweetness balance and texture and these attributes influence repeat purchase intention [18,19]. Drying is the key processing step that ensures shelf stability by lowering moisture and water activity, yet it also drives major quality changes, including browning development, modifications in cellular structure and losses or transformations of volatile compounds [20,21,22]. Notably, the same product category of dried apple snacks can produce very different sensory experiences depending on the drying route and endpoint moisture. Consequently, selecting drying approaches that achieve desirable texture and sweetness perception while preserving nutritional-related attributes becomes central to product success, especially when consumers compare artisanal products with industrial options [16,19,23].

Traditional dehydration approaches such as sun drying and oven drying remain common due to accessibility and relatively low equipment costs [24,25]. Sun drying often involves long processing times and variable environmental conditions, which can intensify oxidative reactions and compromise heat or oxygen-sensitive constituents, including phenolics and aroma compounds [26]. Oven drying offers improved control of temperature and time and may reduce some safety concerns, however, it can promote structural collapse and intensify browning, depending on airflow, slice thickness and drying kinetics [27,28]. In parallel, commercial dried apples reach the market through diverse industrial technologies and formulations, but their processing conditions are frequently undisclosed, which limits interpretability when commercial products serve as benchmarks [29,30].

The literature recognizes that physicochemical properties such as moisture, colour parameters, sugar composition and phenolic content influence sensory outcomes and consumer acceptance [31,32,33]. Even so, many studies focus on instrumental characterization or sensory profiling in isolation or compare methods without connecting consumer responses to measurable drivers (e.g., browning-related metrics, texture indicators and bioactive retention) [10,34,35]. Fewer works directly compare artisanal drying routes with multiple commercial references while simultaneously documenting nutritional-related markers (TPC and antioxidant activity) and consumer-facing attributes (sweetness balance, flavour naturalness and favoured texture) [16,17]. This fragmentation constrains the translation of findings into practical optimization guidelines and highlights the need for integrated evidence linking processing, compositional and structural changes and consumer preference [36,37].

Sensory methods used in food research typically include panels trained in descriptive techniques and consumer-based approaches [38,39]. Trained panels can provide detailed and reproducible attribute characterization and are useful when associating process variables with sensory drivers. However, they require training, are time-consuming and costly and may not fully reflect consumer perception [40]. Consumer hedonic tests directly measure liking and acceptance and are highly relevant for product development decisions, but they are less diagnostic regarding the sensory reasons behind preferences. Combining effective and analytical approaches is often recommended to strengthen interpretation [41]. In response, rapid sensory methods have expanded as practical alternatives that capture consumer-perceived attributes with limited training [42]. Rapid sensory methods have, therefore, gained prominence, particularly the Check-All-That-Apply (CATA) test, which has evolved into a widely used consumer-friendly profiling tool where participants select all applicable descriptors from a predefined list [43,44,45,46]. CATA is a rapid, scalable method that differentiates products via attribute frequencies and, when combined with hedonic ratings and correspondence analysis, relates perceived attributes to liking and instrumental quality [47,48].

While the impact of drying on fruit quality is widely documented, integrated comparisons that simultaneously evaluate physicochemical and bioactive-related parameters together with consumer perception across artisanal drying routes and commercial reference products for the same apple cultivar remain scarce. This gap limits the identification of robust, consumer-relevant drivers of quality and acceptance in dried apple snacks. To address this, the present study provides an integrated assessment of *Royal Gala* dried apple snacks produced by indirect sun drying (SDA), oven drying (ODA) and two commercial drying methods (CCA and CFA). The work combines physicochemical characterization (colour (including browning-related indicators), sugar profile, total phenolic content (TPC) complemented by phenolic profiling and antioxidant activity (AA)) with texture and consumer sensory evaluation. Consumer testing (n = 100) captured CATA and hedonic responses for key dimensions (appearance, aroma, sweetness, texture and overall liking), together with consumption intention and attribute frequencies, thereby linking product chemistry and structure to perceived quality. In addition, multivariate sensory mapping via correspondence analysis (CA) supports interpretation of sample-attribute associations based on frequency data. By comparing artisanal drying methods with commercial references, the study identifies key drivers of preference and clarifies how drying methods may enhance or compromise both sensory appeal and bioactive-related properties, providing practical insights to guide process improvements and support the development of dried apple snacks that meet consumer expectations while retaining desirable physicochemical and nutritional characteristics.

## 2. Materials and Methods

### 2.1. Apple Samples

*Royal Gala* apples used in this study were acquired from a local market. Soluble solids content (SSC), titratable acidity (TA), pH, and maturity index (SSC/TA) have been previously reported by the authors [12]. Sun-dried samples (SDA) were prepared using an indirect cabinet sun dryer designed in-house. In contrast, oven-dried samples (ODA) were processed with a Captain Jerky 110 food dehydrator (Klarstein, Berlin, Germany). Before each drying session, empty trays were weighed to ensure accurate measurements. Apples were selected, washed and cored, then sliced into rings with an electric slicer to obtain a uniform thickness of approximately 3.0 ± 0.1 mm. The rings were arranged in a single layer on the trays, and the drying treatments were carried out simultaneously using fruit from the same batch and maturity stage to minimise raw-material variability. For comparison with commercial products, a generic store-brand snack from a major national supermarket (CCA) and a specialized dried food brand (CFA) were included, as both explicitly state the use of *Royal Gala* apples in their ingredient lists.

### 2.2. Physical Analyses

#### 2.2.1. Colour Determination and Browning Index

Colour is a fundamental parameter for consumer acceptance. The values for L*, a* and b* were extracted from the surface of the apple slices with a colorimeter (FRU^®^ WR-10 colorimeter). Value L* was the degree of lightness/darkness, a* was the degree of redness/greenness and b* was the degree of yellowness/blueness. A white ceramic plate calibrated the equipment before each experiment. The measurement of the samples was replicated ten times at randomly selected points.

The browning index (BI) was calculated to determine colour differences on the apple’s surface, and was calculated according to the following formula:

$$BI=\frac{100(x-0.31)}{0.17},\;\mathrm{where}\;x=\frac{a^{*}+1.75L^{*}}{5.645L^{*}+a^{*}-3.012b^{*}}$$

The BI is derived from the L*, a* and b* values obtained from the colour analysis. It quantifies the degree of browning or discoloration of the apple surface [49,50].

#### 2.2.2. Instrumental Texture Analysis

The texture of the apple slices was evaluated using a texture analyser (TA. XTplus; Stable Micro Systems, Godalming, UK). Compression and puncture tests were performed using a load cell with a capacity of 30 kg in 10 slices of each sample reference.

- Compression tests

**Figure 1:**
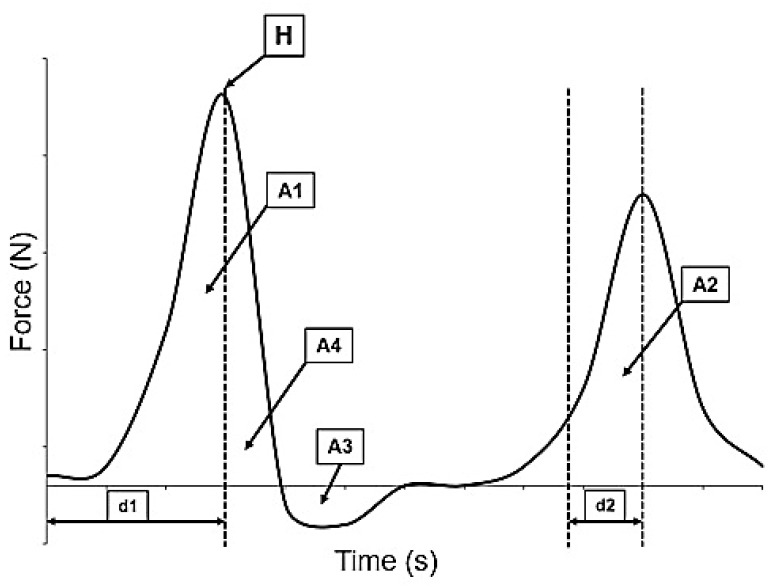
Standard force–time graphic for two-cycle TA.XTplus compression tests: *H*, maximum stress applied on the first cycle; *A*, total area defined by a curve and *d*, time/distance of the probe in each cycle until reaching the maximum force.

In compression tests, two parallel plates were used to compress the samples. It has two cycles and was performed on a known area. A flat aluminium plunger with a diameter of 75 mm (probe P/75) was used. The compression cycles had a set period of 5 s between them at a speed of 1 mm s^−1^, 0.049 N trigger force and 40% strain. The measured parameters provide quantitative information about the textural properties of the apple slices, allowing for a detailed analysis of their compressive behaviour and characteristics: hardness (H), cohesiveness (A2/A1), springiness (d2/d1), chewiness [H × (A2/A1) × (d2/d1)] and resilience (A4/A1). Texture profile parameters were obtained from the force–time curves generated during the compression test (Figure 1) [51].

- Puncture tests

**Figure 2:**
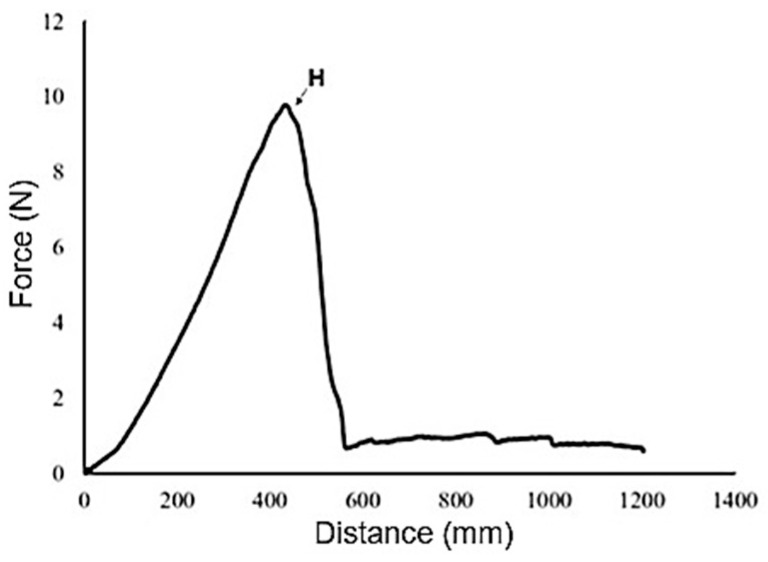
Standard force vs. distance graphic obtained from puncture test.

An 8 mm spherical ball probe was attached to the TA.XTplus of the puncture measurements texture analyser. The heavy-duty platform was aligned so that the ball probe moved precisely through the centre of the crisp support rig, which had a diameter of 1.5 cm. During the test, the probe advanced at a speed of 1 mm/s to penetrate 5 mm into the sample. Hardness was determined as the peak force (N) required to fracture the sample. Each treatment was tested ten times in the TA.XTplus experiments to ensure an adequate sample size for statistical analysis and to achieve reliable results (Figure 2) [51].

The texture parameters were derived from the combined profiles using the EXPONENT v4 software. This software enabled the analysis and extraction of key textural characteristics, offering important information about the texture properties of the various apple slices.

### 2.3. Chemical Analyses

#### 2.3.1. Apple Extracts

The extract was prepared with 1.0 ± 0.01 g of lyophilized and ground dried slices that were weighed and placed in a Falcon tube. A total of 20.0 ± 0.01 mL of extraction solution (80% aqueous methanol acidified with 0.1% HCl) was added and the mixture was vortexed for 2 min. The samples were shaken at 150 rpm for 2 h at room temperature, followed by centrifugation at 3000 *g* for 15 min. The resulting supernatants were used to assess total phenolic content and antioxidant capacity. The same procedure was performed for phenolic profile analysis using a 50% methanolic extraction solution instead.

#### 2.3.2. Determination of Sugars

Soluble sugars were analysed using a high-performance anion-exchange liquid chromatography system with pulsed amperometric detection (HPAEC-PAD). The chromatographic analysis was conducted on a DIONEX ICS 3000 system (Dionex, Sunnyvale, CA, USA) equipped with a quaternary gradient pump and an electrochemical detector featuring a gold (Au) working electrode and an Ag/AgCl reference electrode. Separation was carried out on a CarboPac PA-20 column (30 × 3 mm; Dionex, Sunnyvale, CA, USA), maintained at 30 °C, with a constant flow rate of 0.7 mL/min [52]. All experiments were performed in triplicate.

#### 2.3.3. Determination of Phenolic Compounds

- Total Phenolic Content

The Folin–Ciocalteu colorimetric method generated the standard curve to assess total phenolic content with gallic acid. An aliquot of the extract was combined with 1.0 mL of Folin–Ciocalteu reagent and 7.5 mL of distilled water, followed by vortex mixing. After a 3-min incubation at room temperature, 1.0 mL of 7.5% aqueous sodium carbonate solution was added. The samples were incubated for 90 min at room temperature before measuring absorbance at 765 nm using a Genesys™ 50 spectrophotometer. Absorbance values were compared against the gallic acid standard curve, and the results were expressed as gallic acid equivalents (GAE) in mg per 100 g of apple extract. All experiments were performed in triplicate [53,54].

- Phenolic Profile

A modified version of the method of Golding et al. (2001) [55] was employed to analyse the phenolic profile. High-performance liquid chromatography (HPLC) was used to examine the apple extract. The separation was carried out on a C-18 column (ACE, 250 mm length, 45 mm diameter and 5 μm particle size), maintained at a constant temperature of 35 °C. The mobile phase initially consisted of a 5% aqueous formic acid solution (A) and methanol (B) in a 95:5 (*v*/*v*) ratio for the first 5 min. From 5 to 65 min, the composition was adjusted to 35% (A) and held until 67 min, after which it returned to 95% (A) until 75 min. The flow rate was set at 1.0 mL/min and absorbance was recorded within the 200–600 nm range. Specific wavelengths of 280 nm, 325 nm and 525 nm were used for analysis. Quantification was carried out using a calibration curve based on gallic acid. All measurements were conducted in triplicate.

#### 2.3.4. Antioxidant Activity: DPPH and ABTS Assays

Assessing the antioxidant content in foods is essential, given the growing health risks nowadays. Employing multiple methods is critical to correctly measure a food matrix’s overall antioxidant capacity. The total antioxidant activity of dried apples was evaluated using the DPPH and ABTS methods.

The 1,1-diphenyl-2-picrylhydrazyl radical (DPPH*) scavenging assay assessed the antioxidant capacity. A mixture of 0.1 mL of the dried sample extract and 3.9 mL of freshly prepared DPPH solution (0.06 mM in methanol) was vortexed for 15 s. The solution was then incubated in the dark for 30 min. After incubation, the absorbance was measured at 517 nm, using a spectrophotometer. All experiments were conducted in triplicate [10,56]. The apple samples’ DPPH free-radical-scavenging activity was calculated by the ratio between the absorbance measured in the control and the absorbance measured in the sample.

The free-radical-scavenging activity was measured using the ABTS radical cation decolorization assay. A 7 mM ABTS solution was prepared by dissolving ABTS in water. The ABTS radical was generated by reacting the stock solution with potassium persulfate (2.45 mmol/L) and allowing the mixture to stand in the dark at room temperature for 14–16 h. The resulting solution was diluted with water to absorb 0.700 ± 0.02 at 734 nm. After adding 30 µL of methanol extract to 3.0 mL of the diluted ABTS•+ solution, the absorbance was measured precisely 6 min after the initial mixing. All experiments were performed in triplicate and the results were expressed as μmol Trolox equivalents per g of the apple extract (μmol TE/g) [4,57].

### 2.4. Sensory Analyses

The sensory evaluation of the dried apple samples was carried out following a protocol approved by the Ethics Committee of the Universidade of Trás-os-Montes e Alto Douro (Doc100-CE-UTAD-2024). A consumer study was conducted with 100 participants, recruited through convenience sampling from the university student population, to primarily assess acceptance within this young-adult segment. Participants were included based on their expressed interest in taking part in the project. All participants signed an informed consent form before participating. The session started with an inquiry form with demographic questions, such as gender and age, and clarifications about intolerances and allergies. The evaluations were conducted using a standardized test room (ISO 8589:2007; International Organization for Standardization, 2007, Switzerland) and each session lasted approximately 15 min.

**Figure 3:**
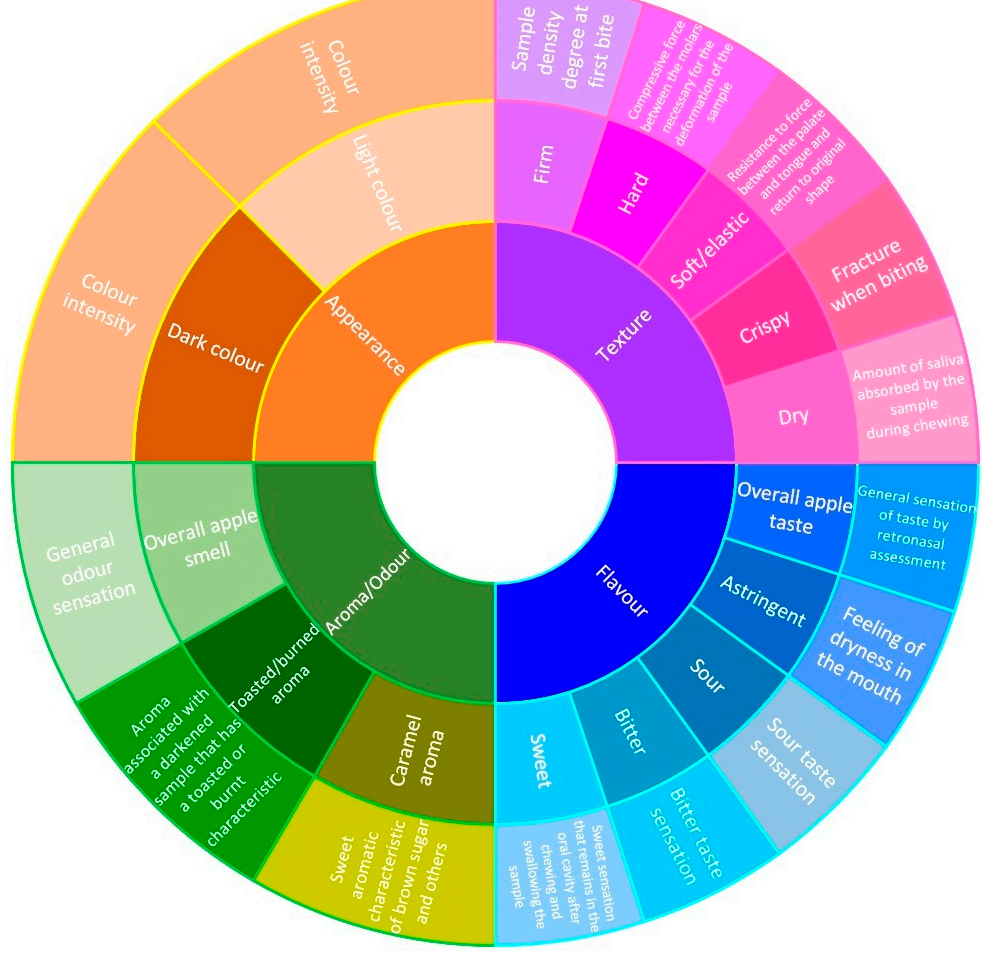
Sensory wheel developed with the attributes selected for the CATA test.

All dried apple samples (SDA, ODA, CCA and CFA) were served simultaneously on white plates divided into four sections, coded with a three-digit reference. Data were collected on mobile phones using an online descriptive questionnaire (Google Forms^®^) with a Check-All-That-Apply (CATA)-type test with 15 descriptors. The descriptors were selected based on the bibliography lexicon of appearance, odour, flavour and texture parameters (Figure 3).

In addition, consumers were also asked to evaluate the products based on each sample’s aroma, appearance, sweetness, texture, overall liking and consumption intention on a 9-point hedonic scale (1 = ‘‘dislike extremely” and 9 = ‘‘like extremely”). All participants were given water to cleanse the palate between samples [58,59].

### 2.5. Statistical Analysis

The data were expressed as mean ± standard deviation. Significant differences (*p* < 0.05) were identified using one-way analysis of variance (ANOVA) followed by Tukey’s post hoc test (unequal *N* when applicable). Statistical analyses were performed using Statistica 12 (StatSoft, Inc., Tulsa, OK, USA). Hedonic sensory ratings were collected using a within-subject design (n = 100; each participant evaluated the SDA, ODA, CCA and CFA samples). Normality of model residuals was assessed prior to ANOVA using the Shapiro–Wilk test and Q–Q plot inspection and the diagnostics supported the use of parametric analysis. Correspondence analysis was performed in IBM SPSS Statistics 27.

## 3. Results and Discussion

### 3.1. Physical Analyses

#### 3.1.1. Colour Analysis (L*a*b*) and Browning Index

The colour of apple pulp diverges across the different drying processes [60,61]. The change in colour of dried fruit results from physical, chemical and biological reactions that occur during the thermal processing of the samples and drying temperature may affect the alteration or degradation of the bioactive compounds [62]. The results of colour analyses are shown in Table 1.

Table 1 shows representative images of the dried samples under study. The colour differences are visually perceptive, which could occur due to the different types of drying that might stimulate most of the enzymatic and non-enzymatic reactions. However, we are unaware of the commercial snacks’ drying conditions. Other parameters beyond temperature can influence the final colour, like the one described by Albertos et al. [63] and Mariscal and Bouchon [64] relating to pressure. Also, fruit acidity can modulate browning kinetics by influencing polyphenol oxidase activity. However, the SDA and ODA samples were produced from the same purchased batch, so initial acidity was constant across these treatments and cannot account for the BI differences between them [65,66]. For commercial products, the initial acidity and pre-treatments are not disclosed, which may contribute to variability [41,42].

The browning index represents the purity of brown colour associated with forming brown pigments that lead to physical and organoleptic changes [49,67]. Browning is accompanied by a decrease in L* and an increase in a* and b* values [68] that is translated in BI, with 96.78 ± 2.3, 161.83 ± 3.5, 192.62 ± 1.2 and 194.10 ± 3.7 for the SDA, ODA, CCA and CFA samples, respectively. Besides enzymatic browning, colour alterations can occur because of non-enzymatic browning; Maillard reactions that induce the appearance of dark spots and burnt taste are related to chemical composition alterations [16].

#### 3.1.2. Instrumental Texture Analysis

**Figure 4:**
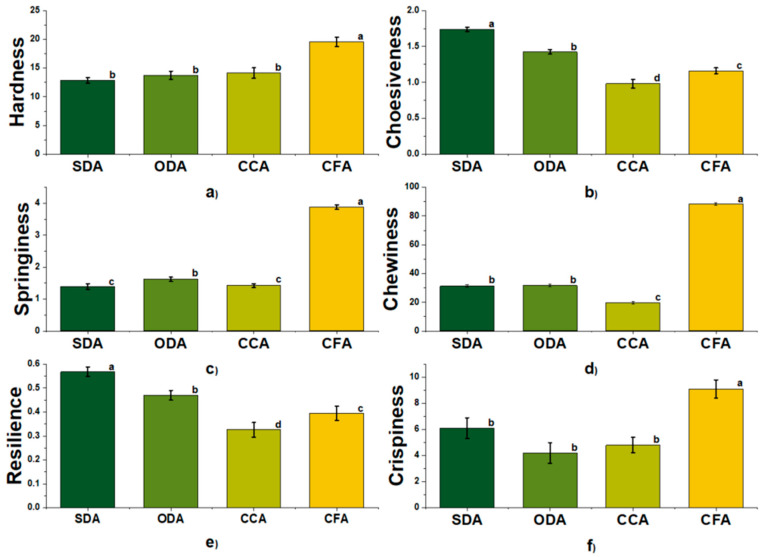
TAXT parameters analyses ((**a**)—hardness; (**b**)—cohesiveness; (**c**)—springiness; (**d**)—chewiness; (**e**)—resilience and (**f**)—crispiness) of dried apples SDA, ODA, CCA and CFA. Data are expressed in arbitrary units as mean ± standard deviation (n = 10). Values with the same lowercase letters in the same parameter are not significantly different (*p* > 0.05). SDA—sun-dried apples; ODA—oven-dried apples; CCA—national supermarket apples and CFA—specialized-dried-food-brand apples.

Texture is a principal quality parameter that consumers use to determine the acceptability of dried food products. Its characteristics can vary according to the different processing techniques. The results of TAXT analyses are shown in Figure 4:

The results obtained in Figure 4a–e were acquired with a P/75 probe. The SDA, ODA and CCA samples exhibit moderate hardness and are significantly different (*p* < 0.05) from the CFA sample, which has the highest level. The registered differences may reflect the combined effects of processing conditions and product matrix characteristics rather than being due to a single identifiable mechanism that could result in a softer texture [10].

The highest level of cohesiveness was registered in the SDA samples, which can result from retained internal structure due to slower drying, which preserves cellular integrity. However, all the samples are significantly different (*p* < 0.05). In general terms, lower cohesiveness is associated with more brittle structures and a more fragmented internal matrix, which can be desirable for crispy snack textures but may reduce chewability [69]. Springiness values were lower for the SDA and CCA samples and significantly different from the ODA and CFA samples, which also differed from each other (*p* < 0.05). Among the four products, the CFA samples exhibited the highest springiness, indicating greater elastic recovery after deformation relative to the other samples. The low springiness reflects a drier, less elastic structure standard in traditional commercial products [70].

Chewiness was highest for the CFA samples, followed by the SDA and ODA samples, while the CCA samples showed the lowest value. As chewiness is mathematically influenced by hardness, cohesiveness and springiness, this pattern is consistent with the CFA samples combining higher springiness with higher resistance to deformation, whereas the CCA’s lower chewiness reflects lower values in one or more of these contributing parameters. The SDA samples showed the highest resilience, followed by the ODA and CFA samples, with the CCA samples presenting the lowest value. Higher resilience indicates greater recovery immediately after compression under the test conditions.

The crispiness parameter, which was evaluated with puncture tests, is highest in the CFA samples, followed by the SDA, CCA and ODA samples, showing significantly lower levels (*p* < 0.05). These results suggest that the CFA samples were designed to be crispy. For the SDA, CCA and ODA samples, the lower crispiness corresponds to less structural damage, making these suitable for consumers who prefer chewy rather than crispy textures [71].

### 3.2. Chemical Analyses

#### 3.2.1. Sugars

**Figure 5:**
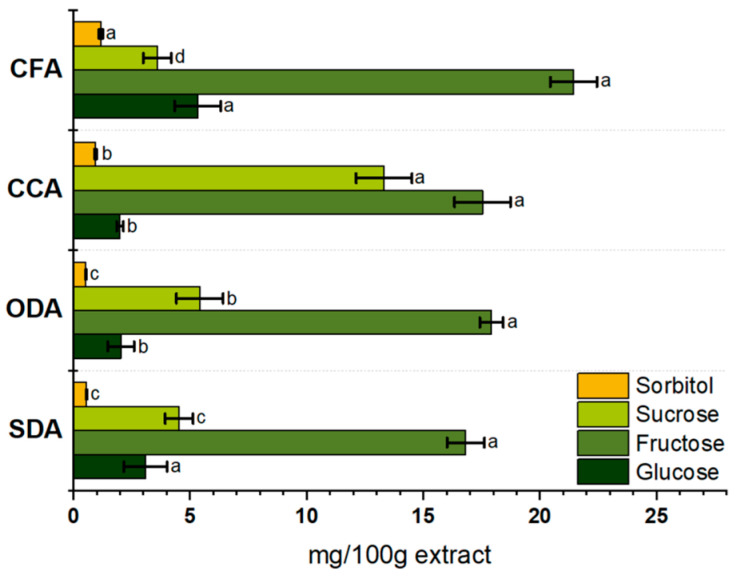
Sugars content (glucose, fructose and sucrose) and sorbitol of dried apples SDA, ODA, CCA and CFA. Data are expressed in mg of sugar/100 g of dried apple extract as mean ± standard deviation (n = 3). Values with the same lowercase letters in the same sugar are not significantly different (*p* > 0.05). SDA—sun-dried apples; ODA—oven-dried apples; CCA—national supermarket apples; CFA—specialized-dried-food-brand apples.

Sweetness is one of the leading indicators of consumer preferences. In this study, we measured the primary sugars (sucrose, glucose and fructose), along with sorbitol and investigated their impact on the perceived sweetness of apples. The amount of sugar present in dried samples is reported in Figure 5.

The total sugar content in the analysed samples, which is the sum of the quantified sugars fructose, glucose, sucrose and sorbitol, ranged from 25.0 to 33.8 mg per 100 g of dried-apple extract. Fructose is the predominant sugar in all the samples, supporting the sweetness-related sensory responses, once sugars may also contribute to mouthfeel and perceived texture through matrix plasticization, notably via sorbitol [72].

For the SDA samples, fructose represents 67.3%, sucrose 18.1% and glucose 12.4% of the total sugars determined. The ODA samples have 69.1% fructose, 20.9% sucrose and 7.9% glucose. For the SDA and ODA samples, sorbitol has low expression (0.57 and 0.54 mg/100 g of extract, respectively).

In the CCA samples, fructose represents 51.9%, sucrose 39.4% and glucose 5.9%. The figures for the CFA samples are 68.0% of fructose, 16.9% of glucose and 11.4% of sucrose. In both commercial samples, the CCA and CFA, the sorbitol level was found to be 2.8 and 3.7%, respectively. Fructose was the most prevalent sugar in all the tested samples, followed by sucrose, glucose and sorbitol. Similar results were found by Wu et al. [73] and Aprea et al. [74]. Sorbitol and glucose are the leaves’ photosynthesis products that reach the fruit tissues by phloem. They are converted into fructose, glucose, malic acid, or starch. In apples, just a tiny part of fructose is incorporated into starch, and the most considerable fraction is accumulated in vacuoles, which validates our results. Sorbitol is the prominent sugar alcohol in apples [40,41], is considered essential to perceive sweetness, and is related to consumer acceptance more than any other single sugar [75,76]. Despite the natural presence in some fruits, sorbitol is also commercially used to preserve moisture, add sweetness and provide texture to products, although it can be associated with gastrointestinal disorders [77].

In summary, the solar- and oven-drying processes reduce glucose and sucrose levels, possibly due to heat-induced caramelization or hydrolysis [78]. Fructose appears to concentrate during water removal, maintaining its dominance in dried samples [79]. The commercial samples exhibit distinct sugar profiles.

The CCA samples showed comparatively higher sucrose levels, which may reflect product-to-product variability, including differences in raw material and/or processing conditions; however, the available label information does not allow inferences regarding sucrose addition. For the CFA samples, the measured sugar profile indicates a different compositional pattern relative to the other samples, although the underlying technological causes cannot be determined from the present dataset. Beyond the dehydration route, factors such as cultivar, growing origin and ripeness can also influence the sugar levels in the final products [80].

#### 3.2.2. Phenolic Compounds

- Total Phenolic Content

**Figure 6:**
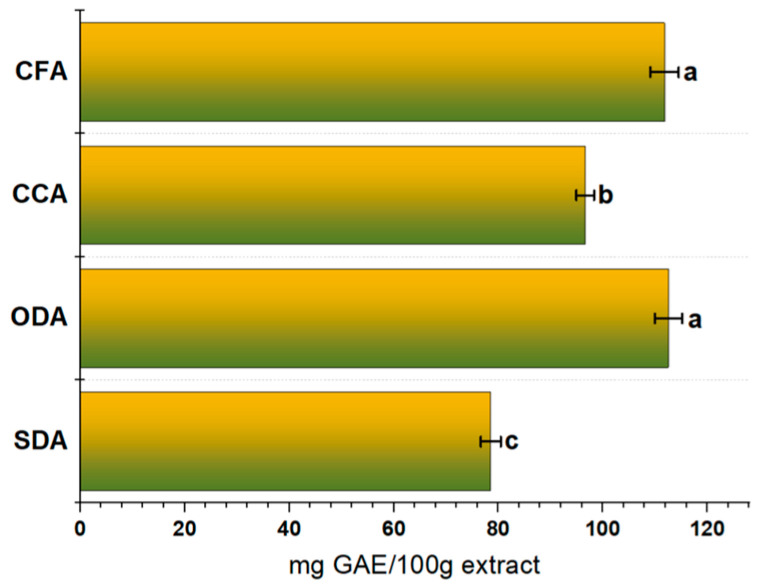
Total phenolic content (TPC) of dried (SDA and ODA) and commercial samples (CCA and CFA). Data are expressed as mean plus/minus standard deviation (n = 3). Values with the same lowercase letters are not significantly different (*p* > 0.05). SDA—sun-dried apples; ODA—oven-dried apples; CCA—national supermarket apples and CFA—specialized-dried-food-brand apples.

The total phenolic content (TPC) of whole apple fruit under different drying techniques was determined using the Folin- Ciocalteu method. Obtained results are shown in Figure 6.

The ODA samples presented the highest TPC among all the samples (112.5 ± 2.6 mg GAE/100 g of apple extract), followed by the CFA samples (111.8 ± 2.7 mg GAE/100 g of apple extract) and the CCA samples (96.6 ± 1.7 mg GAE/100 g of apple extract). The SDA samples had the lowest phenolic retention (78.6 ± 1.9 mg GAE/100 g of apple extract), probably due to the oxidation processes occurring during the exposure time in the drying period.

Different thermal-drying methods might originate from the increased activity of enzymes, causing the degradation of some phenolic compounds. The levels of TPC indicated that drying conditions significantly affected the final products once the values diverged among the samples under study. Lammerskitten et al. [81] described in their research with mangoes that gentle drying can decrease the material’s temperature and improve TPC retention. In several studies, it has been demonstrated that, during drying processes, the browning that has formed on the products may react with Folin’s reagent and explain the alterations in the TPC content in dried apples [82,83,84]. The microstructure of *Royal Gala* apple slices can also influence the retention levels of phenolic compounds. Higher temperatures during drying can lead to cell-wall damage, moisture loss and tissue collapse, causing phenolic compounds to degrade or become less bioavailable [85].

Dried apples’ total phenolic content (TPC) can vary significantly due to drying method, temperature and duration [86]. For example, with hot-air drying (HAD), phenolic degradation is more pronounced due to the high temperatures (usually above 60 °C), leading to a loss of antioxidant properties. On the other hand, freeze drying (FD), which removes moisture by sublimation at low temperatures, helps maintain higher phenolic content because it minimizes heat exposure and oxidation [26]. Air or oven drying at higher temperatures may result in a more substantial loss of phenolic compounds due to heat-induced degradation [87].

Phenolic compounds play a key role in terms of apples’ antioxidant properties and often degrade when exposed to heat and air during processing. These differences may be related to the fresh apple samples’ growing conditions and maturity [88,89].

- Phenolic Profile

**Figure 7:**
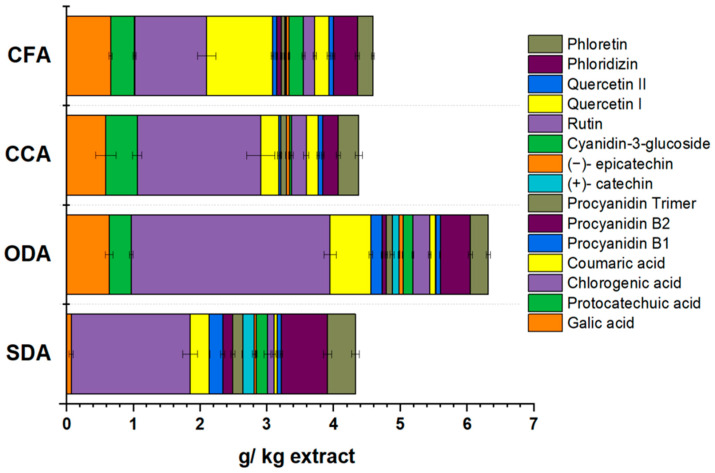
Phenolic profile of dried (SDA and ODA) and commercial samples (CCA and CFA). Data are expressed as mean plus/minus standard deviation (n = 3). SDA—sun-dried apples; ODA—oven-dried apples; CCA—national supermarket apples; and CFA—specialized-dried-food-brand apples.

To further explore the influence of different drying methods on the retention of individual phenolic substances, the phenolic compounds were identified and quantified by HPLC, as shown in Figure 7.

Based on our results, all the analysed samples contained the same phenolic compounds. We identified six distinct classes of phenolics in a total of 15 compounds: hydroxybenzoic acids (gallic acid and protocatechuic acid), hydroxycinnamic acids (chlorogenic acid and coumaric acid), flavanols (procyanidins and catechins), anthocyanidins (cyanidin-3-glucoside), flavonols (rutin and quercetins) and dihydrochalcones (phloridizin and phloretin).

Chlorogenic acid is dominant in all samples. Similar findings were reported by Juhart et al. [90] and Wojdylo et al. [4]. Its levels vary slightly among samples, with the ODA showing higher retention, suggesting that this drying method may preserve it better. Together with coumaric acid, they form the hydroxycinnamic acid group. The ODA samples registered the most significant amount, with 3.59 g/kg apple extract, followed by the CCA, SDA and CFA samples, with 2.12, 2.07 and 1.41 g/kg apple extract, respectively.

The hydroxybenzoic acid group includes protocatechuic acid and gallic acid. Protocatechuic acid shows the highest levels in the CCA samples (0.47 g/kg apple extract) but drops in the CFA and ODA samples (0.36 and 0.33 g/kg apple extract) and is inexistent in the SDA samples. Gallic acid is relatively stable across the samples, though lower in the SDA samples. The processing methods slightly affect the hydroxybenzoic acid content, with the CCA samples showing higher degradation resistance.

Total procyanidins include procyanidin B1, procyanidin B2 and procyanidin trimer. Procyanidins are highest in the SDA samples (0.50 g/kg apple extract), the ODA samples retained a moderate level (0.32 g/kg apple extract) and the CFA/CCA samples have lower procyanidin content, suggesting high sensitivity to commercial processing methods.

The (+)-catechin and (−)-epicatechin dominate the group of total flavanols. The SDA samples have the highest flavanol content (0.70 g/kg apple extract), with the ODA samples retaining a significant portion (0.48 g/kg apple extract). The CCA and CFA samples retain moderate amounts, but the CCA samples consistently underperform, likely due to harsher processing conditions.

Only cyanidin-3-glucoside was detected among anthocyanidins. All samples have similar levels, though the CFA and SDA have slightly higher amounts (0.22 and 0.17 g/kg apple extract), followed by the ODA (0.15 g/kg apple extract).

The flavonols group includes rutin, quercetin I and quercetin II. The CCA samples have the highest flavonol content (0.47 g/kg apple extract), followed by the CFA (0.45 g/kg apple extract) and the ODA samples (0.41 g/kg apple extract). The SDA samples show the lowest retention (0.21 g/kg apple extract), likely due to the impact of the solar drying method.

Phloridizin and phloretin are included in the dihydrochalcone group. The SDA samples have the highest dihydrochalcone content (1.11 g/kg apple extract), followed by the ODA (0.72 g/kg apple extract). The CFA (0.59 g/kg apple extract) and CCA (0.54 g/kg apple extract) samples show the lowest values, confirming that commercial methods degrade these compounds the most.

Summing up all groups, the ODA samples preserve the highest total level of polyphenols (6.32 g/kg apple extract) among the drying methods, followed by the CCA (4.38 g/kg apple extract), SDA (4.33 g/kg apple extract) and the CFA samples (3.93 g/kg apple extract), showing that these commercial methods significantly reduce polyphenol content.

The phenolic compounds identified contribute to the antioxidant capacity of apple products. The sample variation likely reflects differences in drying methods or apple treatment, as processing conditions influence phenolic content.

#### 3.2.3. Antioxidant Capacity Assays

**Figure 8:**
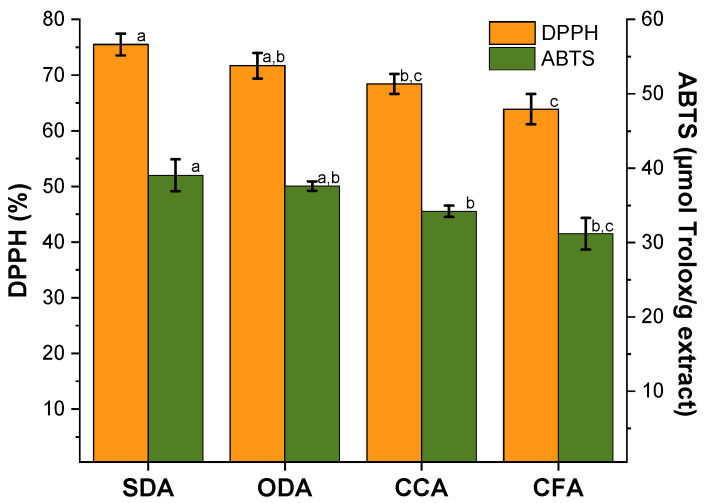
Antioxidant activity (AA) of dried (SDA and ODA) and commercial samples (CCA and CFA). Data are expressed as mean plus/minus standard deviation (n = 3). Values with identical lowercase in the same test letters are not significantly different (*p* > 0.05). SDA—sun-dried apples; ODA—oven-dried apples; CCA—national supermarket apples and CFA—specialized-dried-food-brand apples.

Figure 8 shows the antioxidant activity of tested dried samples measured by DPPH and ABTS free-radical scavenging.

The DPPH-radical-scavenging inhibition of dried apples has demonstrated that the samples follow the order SDA > ODA > CCA > CFA, with values of 75.7, 71.7, 68.4 and 63.9%, respectively.

The SDA samples displayed the highest DPPH antioxidant activity, indicating excellent retention of antioxidant compounds. The level for the ODA samples is slightly lower than for the SDA but still high. It is not significantly different from that for the SDA samples but is better than that for the CCA or CFA. The CCA samples presented lower DPPH activity, denoting a significantly reduced activity compared to the SDA samples. The CFA samples have the lowest DPPH activity, possibly due to the absence of concentration effects during drying.

For the ABTS assay, the SDA samples maintained the trend showing the highest activity (39.0 ± 2.1 µmol Trolox/g apple extract), comparable to DPPH performance, indicating it retains antioxidants effectively. The ODA samples registered slightly lower ABTS values (37.6 ± 0.6 µmol Trolox/g apple extract), though still statistically like the SDA (“a, b”). The CCA samples have 34.2 ± 0.8 µmol Trolox/g apple extract, statistically lower than the SDA but higher than the CFA (“b”). The CFA samples exhibited the lowest ABTS values (31.2 ± 2.1 µmol Trolox/g apple extract). Similar to DPPH, this suggests a lower antioxidant capacity.

The SDA and ODA samples are statistically similar in terms of antioxidant activity. The CCA samples differ statistically from the SDA samples in terms of DPPH and ABTS but overlap with the ODA samples. The levels for the CFA samples are significantly lower than those for the SDA and ODA samples but overlap with those for the CCA samples in the ABTS assay. Higher DPPH values in the SDA and ODA samples reflect the preservation of phenolic compounds. The discrepancies between DPPH and ABTS results suggest that the drying methods affect the conservation of antioxidant compounds differently.

Solar drying is the most effective drying method for preserving antioxidant activity, as shown by its performance in both assays. This proved to be a suitable method for producing high-quality dried apple snacks. Oven drying offers a good alternative, with a slight reduction in antioxidant retention compared to the SDA samples. In both antioxidant activity evaluations, the CCA and CFA samples show the lowest antioxidant activity per dried apple extract.

Combining both assays provides a more comprehensive understanding of antioxidant activity in the samples.

### 3.3. Sensory Analyses

#### 3.3.1. Consumers Demographic Data Analysis

The participants were predominantly Portuguese (n = 94), and the remaining nationalities included Brazilians (n = 3), Chinese (n = 2) and Spanish (n = 1), which may introduce some cultural influence on the perception of apple snacks. Most participants (88%) were young adults aged 18–25, which may not fully represent the broader market, however, this is a key demographic group for understanding snack preferences and purchasing trends. Nevertheless, the use of standardized sensory conditions, blind three-digit coding and palate cleansing supports robust within study comparisons among samples. The gender distribution was 66% female and 34% male, allowing insights into male and female perspectives. Regarding apple snacks, in general, most participants viewed apple snacks positively (64%), although 33% had no opinion, highlighting the need to increase awareness and consumer acceptance. A small minority reported intolerances or allergies (9), which suggests minimal concerns regarding allergen-related barriers for apple snack tests. Only four participants reported health issues or had diseases, indicating that the group is generally healthy. Most participants were not taking medication, further reinforcing the general good health of the group. Most (81 participants) perceive themselves to be in good health. Most participants reported regular habits, namely caffeine, alcohol, chewing gum and tobacco, which may influence snack preferences and consumption behaviour. Insights from this group can help target young, health-conscious consumers for the apple snack products.

#### 3.3.2. Apple Snacks Sensory Evaluation by CATA Test

CATA is nowadays used in consumer research to describe the sensory perception of different kinds of products such as ice cream [58]; apple juice, fruit yogurt, milk chocolate, dried apricots and salted potato chips [44]; fish products [91]; pumpkin [39]; mandarins [46]; biscuits [92]; rice [43]; red wine [93]; kiwifruits [94]; tea [95]; and guavas [96]. It is a rapid and effective technique for gathering consumer insights into sensory characteristics, preferences and product differences.

Table 2 shows the data obtained by the CATA technique, with defined characteristics that respond to consumers’ needs.

According to the CATA test results, in terms of appearance, light colour was more frequent in the SDA samples (80), indicating solar drying preserves lighter coloration effectively. A dark colour was present in the ODA (53), CCA (50) and CFA (46) samples, probably due to browning or caramelization from high-temperature drying [97]. In the aroma/odour group of attributes, apple odour is more prominent in the SDA samples (41), followed by the ODA (30), and is lowest in the CFA (23) and CCA (18) samples; possibly, the drying process might influence the retention of the fresh apple odour. The toasted/burned aroma is most noticeable in the ODA samples (32), indicating a tendency for oven drying to produce these notes [98]. The same tendency was registered for the caramel attribute (36).

In the flavour group, sweetness was most perceived in the SDA (84) and ODA (74) samples but was lower in the CCA and CFA (57) samples, indicating differences in sugar retention or perception. Bitterness was generally low across all the samples, although it was higher in the commercial samples (19 in the CCA > 18 in the CFA > 15 in the ODA > 7 in the SDA). Acidity had the highest frequency in the CCA samples (31) and the lowest in the CFA samples (18), potentially reflecting differences in drying methods affecting acid perception. Astringency had a minimal impact and there was very low occurrence across all the samples. The overall apple taste was highest in the SDA samples (80), reinforcing its strong sensory retention, and declined in the ODA (63), CFA (53) and CCA (50) samples. Regarding the texture, dryness is least present in the SDA (30), ODA (35) and CCA (41) samples, suggesting their less drastic moisture removal process and was most noted in the CFA samples (51).

Crispiness was less frequent in the CCA samples (63) and most noted in the ODA samples (84), indicating that oven drying preserves a crunchier texture. The CCA (24) samples were also chosen as the softer/elastic snacks, corroborating this tendency.

The most challenging parameter involved the CFA samples (31), aligned with the structural alteration of the samples, which became denser with the drying process. Firmness results were not consensual among the participants for the SDA (34), ODA (36), CCA (26) and CFA (27) samples.

**Figure 9:**
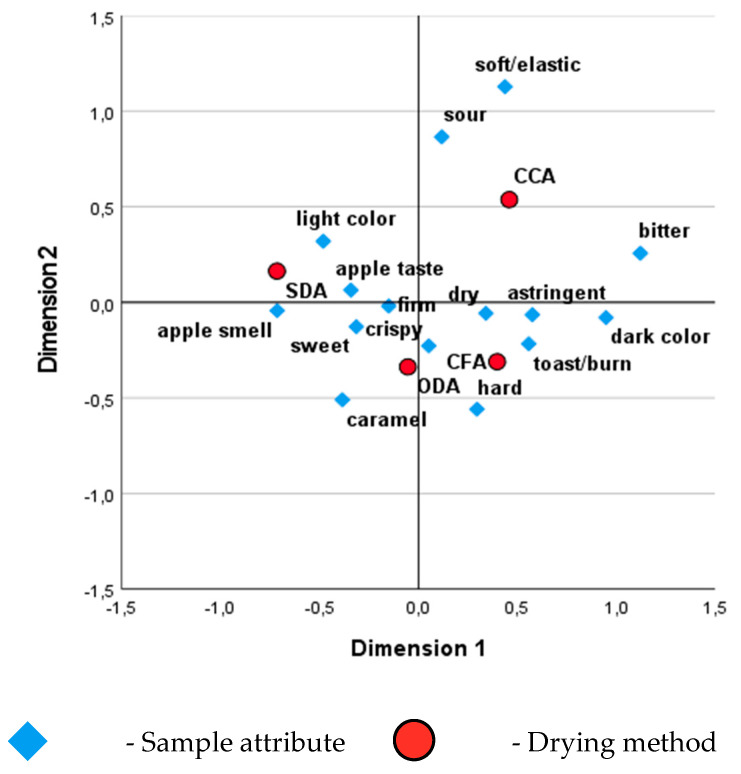
Correspondence analysis between categorical variables drying method vs. sample attribute. SDA—sun-dried apples; ODA—oven-dried apples; CCA—national supermarket apples and CFA—specialized-dried-food-brand apples.

A correspondence analysis was made with SPSS (Figure 9) to study the relation between drying methods and the studied attributes, with the variables having discrete categories rather than numerical values.

A red-coloured point represents each sample type (drying method) and its proximity to specific attributes reflects the sensory characteristics most closely associated with that sample.

The SDA samples are positioned close to positive attributes such as “sweet,” “apple smell,” “light colour,” and “apple taste.” That indicates they are perceived as flavourful and appealing, likely due to their natural sweetness and aroma retention. The ODA samples are located near “crispy,” “firm,” “dry,” and “hard” attributes, which suggests they are associated with desirable textural qualities, such as crispiness and firmness, which some consumers may prefer. Concerning the CCA samples, they are positioned further away from most positive sensory attributes, closer to “sour” and “bitter.” It indicates a less favourable sensory profile, as the association with bitterness and sourness could reduce consumer appeal. The CFA samples are located near attributes such as “crispy,” “toast/burn,” and “dark colour.” That suggests these samples may have undergone processes emphasizing browning and toasted notes. While some consumers may enjoy toasted flavours, the association with “dark colour” could also imply over-processing or burning.

#### 3.3.3. Apple Snacks’ Hedonic Perception

**Figure 10:**
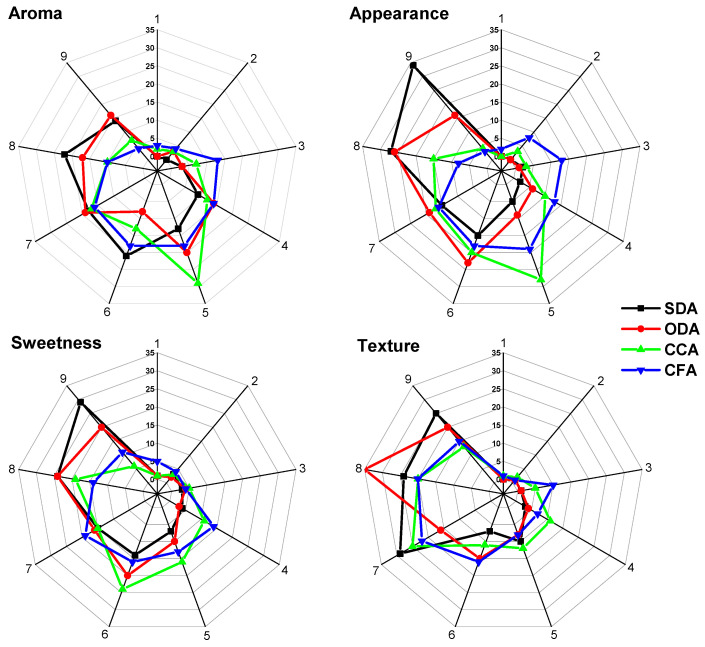
Spider plots show how participants evaluated the tested products in terms of aroma, appearance, sweetness and texture. SDA—sun-dried apples; ODA—oven-dried apples; CCA—national supermarket apples and CFA—specialized-dried-food-brand apples.

Spider plots (Figure 10) were made to compare sensory attributes across the SDA, ODA, CCA and CFA samples based on a 1–9 hedonic scale, where 1 = dislike extremely and 9 = like extremely.

The axis of the spider plots shows the score from the hedonic scale, which indicates how each sample is perceived and how positively it is evaluated for that specific attribute. The SDA samples show strong evaluation in terms of all the attributes, the ODA samples have consistency in median scores, the CCA samples’ strengths are more linked to texture and the CFA samples have the lowest scores, especially for the aroma. All the results follow the correspondence analyses.

Focusing on the SDA samples’ attributes. Scores indicate they meet consumer expectations in terms of aroma, texture, sweetness and flavour. The ODA samples could be enhanced by sweetness or texture to increase their appeal further. It would be interesting to re-evaluate the CCA and CFA production process to align with consumer preferences. Adjustments in texture, sweetness, or aroma may improve their acceptability.

**Figure 11:**
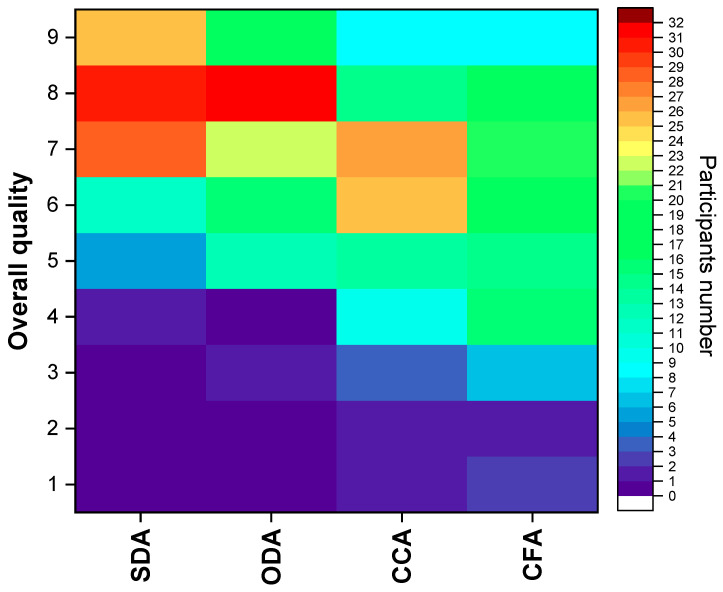
Heat map of the overall quality of each dried product: SDA, ODA, CCA and CFA. SDA—sun-dried apples; ODA—oven-dried apples; CCA—national supermarket apples and CFA—specialized-dried-food-brand apples.

Regarding the overall quality of each product, the results are represented in a heat map that visually represents the scores for the four samples (the SDA, ODA, CCA and CFA) based on a 1–9 hedonic scale (Figure 11).

The SDA samples have the lowest scores, above 5, associated with the colour, with fewer participants. A total of 11 participants’ attribution score was 6 and the rest considered the quality equal or superior to 7 (83 participants). The heat map is dominated by orange/red zones, with 58 participants’ choosing scores of 7 and 8. A total of 25 participants scored the samples with a classification of 9.

For the ODA samples, scores between 5 and 9 were chosen by almost all the participants (99). This suggests moderate acceptance but an appeal to a subset of consumers who gave it high scores (8 and 9 with 32 and 18, respectively).

With the CCA samples, 26 participants chose scores ranging from 2 to 5, with 7 being the most selected (26 participants). This suggests that this sample has moderate to low acceptance levels, indicating a more controversial product profile. The CFA samples gave rise to a more divided general opinion, with 38 participants giving a score of 5 or minus and 54 participants choosing between 6 and 8. This is the least favoured product and has the lowest overall acceptance.

In conclusion, the SDA and ODA samples were the best-performing samples, with the highest top ratings (8–9) and almost no low scores. The CCA and CFA samples received mostly moderate ratings (4–6), indicating they were generally accepted but not preferred. Typically, participants prefer the SDA and ODA samples, finding the CCA or CFA samples acceptable.

#### 3.3.4. Apple Snacks’ Hedonic Acceptability

**Figure 12:**
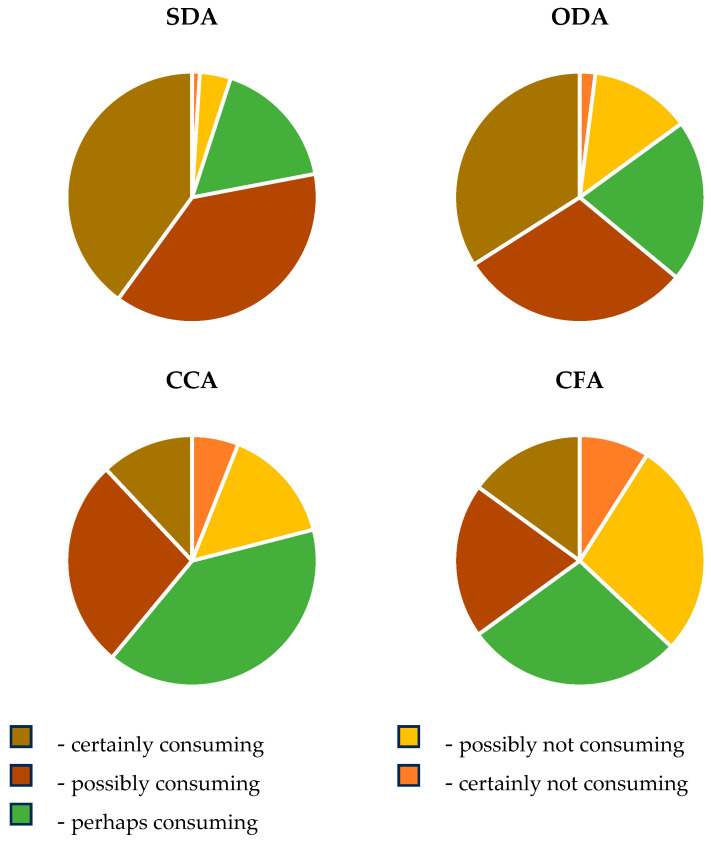
Consumer preferences for each dried product: SDA, ODA, CCA and CFA. SDA—sun-dried apples; ODA—oven-dried apples; CCA—national supermarket apples and CFA—specialized-dried-food-brand apples.

The consumer preference trends across samples are shown in Figure 12.

According to our inquiry results, the SDA samples have the highest consumer acceptance, with 38 individuals possibly consuming them and 40 certainly consuming them. They are the least rejected, with just one respondent saying, “certainly would not consume it”. The combination of “possibly” and “certainly” consumes scores (78 total) makes them the most favourable option overall.

The ODA samples are similar in terms of acceptance to the SDA samples but slightly less favourable, with 34 respondents saying they “certainly would consume it”, and 30 respondents saying they would “possibly consume it”, which totals 64 for potential consumption. Rejection rates were low, with only two respondents saying they would “certainly not consuming it” and 13 saying they would “possibly not consuming it.” The commercial CCA samples generated more balanced responses, but participants showed less enthusiasm overall. Only 12 respondents said they “would certainly consume it”, and 30 said they “would possibly consume it”, which totals 42 for potential consumption. Rejection is higher than it was for the SDA and ODA samples, with six respondents saying they would “certainly not consuming it” and 15 saying they would “possibly not consuming it”. The CFA samples show the least consumer acceptance, with 15 respondents saying they “would certainly consume it” and 20 saying they would “possibly consume it”, which totals 35 for potential consumption. Rejection rates were the highest, with nine respondents saying they would “certainly not consuming it” and 28 saying they would “possibly not consuming it”.

In conclusion, the SDA samples were the most accepted, as they have the highest number of respondents in the “certainly consume” and “possibly consume” categories. This suggests they may preserve attributes like flavour, texture, or perceived natural qualities that appeal to consumers. The ODA samples occupy an intermediate position, showing significant acceptance but less than the SDA samples. Commercial samples (CCA and CFA) face challenges in terms of consumer acceptance. Both have high rejection rates, and the CFA sample performed the worst, with the lowest positive scores across all categories. The consumer rejection rate was more pronounced for the commercial samples (CCA and CFA), potentially due to differences in taste, texture, or perception of quality compared to the SDA and ODA samples.

#### 3.3.5. Correlation of Texture Sensory Analysis and Consumers’ Acceptance of Dried Apple

Texture is a critical attribute influencing food products’ overall sensory perception and acceptability. For dried apples, texture attributes such as hardness, crispiness, dryness and firmness play significant roles in determining consumer preferences [2]. Samples that exhibited medium crispiness (the SDA and ODA samples) tend to score higher when it comes to consumer preference, particularly on the “possibly” and “certainly” consume scales. This confirms that crispiness positively correlates with overall consumer acceptance of dried apples, but only when the snack does not become too hard [99]. Samples such as the CCA ones, which showed a higher association with “soft/elastic” characteristics correspond to lower acceptance scores. Consumers may perceive soft or elastic textures in dried apples as undesirable, possibly due to improper drying or moisture retention [100]. Moderate firmness appears favourable (the SDA and ODA samples), while excessive firmness (the CCA and CFA samples) may reduce acceptance due to chewiness or toughness.

While a balanced dryness level is desirable, excessive hardness can reduce acceptability [101]. For example, in the case of the CFA sample, which was correlated with attributes like “dry” and “hard,” a lower percentage of consumers were willing to accept the product.

To improve consumer acceptance of dried apples, the drying process should be optimized to achieve textures that balance crispiness and moderate firmness and avoid undesirable softness or excessive hardness [51].

The SDA samples presented with higher cohesiveness, resilience and moderate chewiness, making them suitable for consumers who enjoy softer and natural textures. Their lower crispiness demonstrates that they are better suited for a “healthier snack” profile, emphasizing minimal processing. The ODA samples were similar, but with less resilience and cohesiveness. They balanced chewy and firm textures, appealing to a broader public. These methods are closer to the positive attributes such as sweetness, apple aroma and desirable textural qualities. This aligns with their higher acceptance rates in the intention-to-consume results.

Among the commercial samples, the CCA samples had lower chewiness and crispiness, making them less crispy than the CFA samples, but they were still firmer than the SDA and ODA samples. They are associated with negative sensory traits such as sourness and bitterness, potentially impacting their acceptance. The CFA samples presented high hardness, chewiness, springiness and crispiness, making them suitable for crispy snack lovers. However, low cohesiveness and resilience indicate more brittle structures. They mix positive (caramel) and negative (toast/burn and dark colour) traits, suggesting they may have a niche but come with low overall acceptance.

## 4. Conclusions

Consumer expectations of dried apple snacks as being naturally sweet are consistent with the measured sugar profile, with fructose predominating across products. All samples presented TPC values of approximately ≥80 mg GAE/100 g extract and the same set of 15 identified phenolic compounds, indicating a broadly comparable qualitative phenolic profile, although antioxidant performance differed among products. Across assays, antioxidant activity followed the pattern SDA > ODA > CCA > CFA. From a consumer perspective, the SDA and ODA samples showed the highest acceptance levels, with most participants assigning an overall liking score of 8/9, while exhibiting distinct sensory positioning; meanwhile, the SDA samples were more frequently associated with a lighter appearance and apple-like aroma/flavour cues, whereas the ODA samples aligned with crispiness/firmness and caramel/toasted notes. Commercial references (the CCA and CFA) exhibited different attribute-selection patterns, including sourness/elastic texture for the CCA samples and darker/toasted/astringent cues with a harder texture for the CFA samples. However, because processing conditions and formulation details are not disclosed, these outcomes should be interpreted descriptively rather than as evidence of specific industrial practices. Overall, the integrated physicochemical and consumer sensory results indicate that the drying technique is associated with measurable differences in colour/browning development, antioxidant-related markers and consumer-perceived attributes in *Royal Gala* dried apple snacks. These conclusions apply to the specific products evaluated and to the presented consumer samples. Within this set, the commercial products were generally rated lower in terms of their sensory attributes than the in-house samples. Overall, the results highlight how different drying methods influence consumer-perceived sensory attributes.

## Figures and Tables

**Table 1:** Surface colour measurement and browning index of SDA, ODA, CCA and CFA samples. BI data is expressed as mean plus/minus standard deviation (n = 3, with six replicates each). Values with the same lowercase letters are not significantly different (*p* > 0.05).

	SDA	ODA	CCA	CFA
Sample	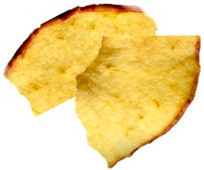	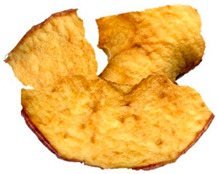	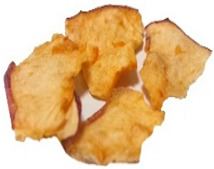	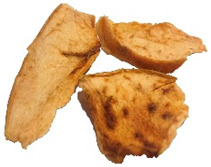
L*a*b*	L*: 84.53 a*: −1.71 b*: 56.12	L*: 75.44 a*: 10.99 b*: 60.24	L*: 64.81 a*: 13.87 b*: 55.17	L*: 57.22 a*: 19.28 b*: 45.29
BI	96.78 ± 2.3 ^c^	161.83 ± 3.5 ^b^	192.62 ± 1.2 ^a^	194.10 ± 3.7 ^a^

SDA—sun-dried apples; ODA—oven-dried apples; CCA—national supermarket apples and CFA—specialized dried food brand apples.

**Table 2:** Frequencies for each descriptor of the check-all-that-apply questionnaire (n/100, where **n** is the number of consumers who checked the descriptor for each sample).

		SDA	ODA	CCA	CFA
**Appearance**	Light colour	80	45	51	50
	Dark colour	18	53	50	46
**Aroma/odour**	Overall apple smell	41	30	18	23
	Toasted/burned aroma	18	32	27	26
	Caramel aroma	29	36	12	20
**Flavour**	Sweet	84	74	57	57
	Bitter	7	15	19	18
	Sour	29	26	31	18
	Astringent	2	4	2	3
	Overall apple taste	80	63	50	53
**Texture**	Dry	30	35	41	51
	Crispy	73	84	63	80
	Soft/elastic	14	9	24	10
	Hard	20	29	22	31
	Firm	34	36	26	27

SDA—sun-dried apples; ODA—oven-dried apples; CCA—national supermarket apples and CFA—specialized-dried-food-brand apples.

## Data Availability

The data presented in this study are available on request from the corresponding authors.
